# Supplementary material for: ProteinShader: illustrative rendering of macromolecules
Source: BMC Struct Biol. 2009 Mar 30;9:19. doi: 10.1186/1472-6807-9-19 (PMC2672931; doi:10.1186/1472-6807-9-19)
Supplement: Additional file 1 — ProteinShader program without source code. This compressed file contains the complete ProteinShader program including associated libraries, but no source code. A README.txt file gives an overview of the ProteinShader distribution, and the index.html file in the help subdirectory has directions on getting started with the program as well as a set of tutorials. [file 1472-6807-9-19-S1.zip › ProteinShader-beta-0_9_4-binary/help/api/org/proteinshader/structure/enums/class-use/AtomEnum.html]

Uses of Class org.proteinshader.structure.enums.AtomEnum (ProteinShader API)


|  |  |  |
| --- | --- | --- |
| **Overview** \| **Package** \| **Class** \| **Use** \| **Tree** \| **Deprecated** \| **Index** \| **Help** | |  |
| PREV   NEXT | **FRAMES**    **NO FRAMES**     **All Classes** |  |


---


## **Uses of Class org.proteinshader.structure.enums.AtomEnum**

| Packages that use AtomEnum | |
| --- | --- |
| **org.proteinshader.structure** | Holds the classes that store information from a Protein Data Bank file: Structure, Model, Chain, AminoAcid, Heterogen, Water, Atom, Bond, Helix, BetaStrand, Loop, *etc*. |
| **org.proteinshader.structure.enums** | Holds the enums needed by org.proteinshader.structure and its subpackages. |
| **org.proteinshader.structure.exceptions** | Holds the exception classes needed by org.proteinshader.structure and its subpackages. |
| **org.proteinshader.structure.io** | Holds the classes needed for reading Protein Data Bank structure files. |
| **org.proteinshader.structure.visitor** | Holds Visitor classes that know how to traverse the hierarchy of objects held by class Structure. |

Uses of AtomEnum in org.proteinshader.structure

| Methods in org.proteinshader.structure that return AtomEnum | |
| --- | --- |
| `AtomEnum` | `Atom.getType()`             Returns the Atom's type as an enum based on the periodic table of the elements. |

| Methods in org.proteinshader.structure with parameters of type AtomEnum | |
| --- | --- |
| `Atom` | `AminoAcid.addNewAtom(int serialNo, AtomEnum type, String atomID, double temperature, int charge, double occupancy, String altLocation, double x, double y, double z, VisibilityEnum visibility)`             Creates a new Atom with the type and Atom ID given as arguments, adds the Atom to the Residue's collection of Atoms, and returns a reference to the new Atom. |
| `Atom` | `Residue.addNewAtom(int serialNo, AtomEnum type, String atomID, double temperature, int charge, double occupancy, String altLocation, double x, double y, double z, VisibilityEnum visibility)`             Creates a new Atom with the type and Atom ID given as arguments, adds the Atom to the Residue's collection of Atoms, and returns a reference to the new Atom. |
| `Atom` | `Structure.addNewAtom(int atomSerialNo, AtomEnum atomType, String atomID, String aminoAcidType, String residueID, String chainID, String modelID, double temperature, int charge, double occupancy, String altLocation, double x, double y, double z, VisibilityEnum visibility)`             Creates a new atom with the atomID given as an argument, adds the new Atom to the requested Model-Chain-AminoAcid, and then returns a reference to the new Atom. |
| `Atom` | `Structure.addNewHetAtom(int atomSerialNo, AtomEnum atomType, String atomID, String hetName, String residueID, String chainID, String modelID, double temperature, int charge, double occupancy, String altLocation, double x, double y, double z, VisibilityEnum visibility)`             Creates a new atom with the atomID given as an argument, adds the new Atom to the requested Model-Chain-(Heterogen or Water), and then returns a reference to the new Atom. |

Constructors in org.proteinshader.structure with parameters of type AtomEnum

`Atom(int serialNo,
AtomEnum type,
String atomID,
String residueID,
String chainID,
String modelID,
String structureID,
double temperature,
int charge,
double occupancy,
String altLocation,
double x,
double y,
double z,
VisibilityEnum visibility)`
  
          This constructor uses the type argument (an AtomEnum) to set the
default radius and CPK color for the Atom.

Uses of AtomEnum in org.proteinshader.structure.enums

| Methods in org.proteinshader.structure.enums that return AtomEnum | |
| --- | --- |
| `static AtomEnum` | `AtomEnum.valueOf(String name)`             Returns the enum constant of this type with the specified name. |
| `static AtomEnum[]` | `AtomEnum.values()`             Returns an array containing the constants of this enum type, in the order they're declared. |

Uses of AtomEnum in org.proteinshader.structure.exceptions

| Methods in org.proteinshader.structure.exceptions that return AtomEnum | |
| --- | --- |
| `AtomEnum` | `BondLengthException.getDstAtomType()`             Returns the destination Atom type as an AtomEnum. |
| `AtomEnum` | `BondLengthException.getSrcAtomType()`             Returns the source Atom type as an AtomEnum. |

Constructors in org.proteinshader.structure.exceptions with parameters of type AtomEnum

`BondLengthException(double calculatedLength,
double expectedLength,
double toleranceFactor,
AtomEnum srcAtomType,
AtomEnum dstAtomType,
int srcAtomSerialNo,
int dstAtomSerialNo)`
  
          Sets the default message to "A Bond length was unacceptable".

`BondLengthException(String message,
double calculatedLength,
double expectedLength,
double toleranceFactor,
AtomEnum srcAtomType,
AtomEnum dstAtomType,
int srcAtomSerialNo,
int dstAtomSerialNo)`
  
          The message given this constructor can be retrieved using
getMessage().

Uses of AtomEnum in org.proteinshader.structure.io

| Methods in org.proteinshader.structure.io that return AtomEnum | |
| --- | --- |
| `AtomEnum` | `PDBAtomFieldExtractor.extractAtomType(String line)`             Obtains the atom type (chemical element) from an ATOM or HETATM record. |

Uses of AtomEnum in org.proteinshader.structure.visitor

| Methods in org.proteinshader.structure.visitor with parameters of type AtomEnum | |
| --- | --- |
| `void` | `Visitor.setMode(AtomEnum atomType)`             Sets the traversal mode with regard to Atom type. |

---


|  |  |  |
| --- | --- | --- |
| **Overview** \| **Package** \| **Class** \| **Use** \| **Tree** \| **Deprecated** \| **Index** \| **Help** | |  |
| PREV   NEXT | **FRAMES**    **NO FRAMES**     **All Classes** |  |


---

# *Copyright © 2007-2008*
